# Exploring the Coordination Effect of GO@MOF-5 as Catalyst on Thermal Decomposition of Ammonium Perchlorate

**DOI:** 10.1186/s11671-019-3163-z

**Published:** 2019-11-21

**Authors:** Shuang Wang, Baoyun Ye, Chongwei An, Jingyu Wang, Qianbing Li, Hao Guo, Jianwei Zhang

**Affiliations:** 1 School of Environment and Safety Engineering, North University of China, Taiyuan, 030051 Shanxi China; 2 Shanxi Engineering Technology Research Center for Ultrafine Powder, North University of China, Taiyuan, 030051 China

**Keywords:** Mental-organic frameworks (MOFs), Graphene oxide (GO), Ammonium perchlorate (AP), Thermal decomposition

## Abstract

Prepared composite materials based on [Zn_4_O(benzene-1,4-dicarboxylate)_3_] (MOF-5) and graphene oxide (GO) via a simple green solvothermal method, at which GO was used as platform to load MOF-5, and applied to the thermal decomposition of AP. The obtained composites were characterized by various techniques such as scanning electron microscopy (SEM), X-ray diffraction (XRD), nitrogen adsorption, Fourier transform infrared (FT-IR), differential scanning calorimetry and thermalgravimetric (DSC-TG). The analyses confirmed that the composite material (GO@) MOF-5 can not only improve the decomposition peak temperature of AP from the initial 409.7 ^°^C to 321.9 ^°^C, but also can improve the enthalpy (△H) from 576 J g^−1^ to 1011 J g^−1^ and reduce the activation energy (*E*_a_), thereby accelerating the decomposition reaction. The high-specific surface area of the MOF material can provide a large number of active sites, so that the transition metal ions supported thereon can participate more effectively in the electron transfer process, and GO plays its role as a bridge by its efficient thermal and electrical conductivity. Together, accelerate the thermal decomposition process of AP.

## Introduction

Combustion performance is an important indicator to measure the performance of a solid propellant. The thermal decomposition properties of the ammonium perchlorate (AP) which is often used as a strong oxidant, plays an important role in combustion performance of the whole propellant composition. Adding burning rate modifiers which will be called further as the catalyst to the propellant formulation can effectively improve its performanc e[1–3]. Especially some metals and their oxides, such as copper [4–9], nickel [10, 11], zinc [12, 13], cobalt [14, 15], and carbon nanomaterials [16–18].

Metal-organic frameworks (MOFs) materials have been widely used in adsorption, catalysis, and optoelectronic devices due to their large specific surface area, high porosity and other excellent properties, and have received great interest in recent years [19–24]. MOFs are formed by self-assembly of metal ions or metal clusters and organic ligands, and generally formed by solvothermal methods under mild conditions in which preparation process is economical and widely used. And these kind of materials have high activity in catalyzing the oxidation of alkanes, olefins, alcohols, and CO, besides, MOFs provide a large number of uniformly dispersed active sites for transition metals, which can generate metal oxides in situ during propellant combustion and promote burning [25]. Hence, based on the above excellent properties, it is possible to use the MOF material in the AP propellant system and use it as a burning rate modifier for the AP to improve the combustion performance of the entire propellant system.

However, the open skeleton structure of MOFs without strong non-specific adsorption and with poor dispersion. For this reason, some scholars try to combine it with carbon nanomaterials, Kumar found that hybrid nanocomposites of GO with ZIF-8 exhibited tunable nanoscale morphology and porosity, and this composite has a high storage rate of CO_2_ [26]; Jabbari developed a hybrid nanocomposites based on Cu-BTC MOF, GO, CNT, and Fe_3_O_4_ magnetic nanoparticles via a simple green solvothermal method, at which GO and CNT were used as platforms to load nanostructured Cu-BTC MOF and Fe_3_O_4_ MNPs and the hybrid nanomaterials showed enhanced pollutant adsorption capacity compared to that of the parent materials [27]; Ge Chunhua prepared carboxyl-rich carbon spheres (CCSs) and copper-based metal–organic frameworks (MOFs), and it is disclosed that carboxyl-rich groups of the CCSs could substantially enhance the connection with the Cu ion in MOF, and benefit for the homogenous surface growth of HKUST-1 on the core of CCSs [28]. Many studies have shown that the rich functional groups on the surface of GO make it a potential platform to promote the growth of MOF and enhance its dispersing power. At the same time, the presence of GO can effectively improve the electrical and thermal conductivity of the composite.

In this work, a composite of MOF-5 and GO was prepared by the green solvothermal method and added to AP to investigate its catalytic effect on thermal decomposition of AP.

## Methods

### Materials

Ammonium perchlorate (NH_4_CIO_4_, AP) powder was purchased from Sinopharm Chemical Reagent Co., Ltd. Reagent-grade Zn(NO_3_)_2_·6H_2_O, 1,4-benzenedicarboxylic acid (H_2_BDC), ethyl alcohol, DMF, and ethyl acetate were purchased from Tianjin Guangfu Technology Development Co., Ltd. GO was prepared with modified Hummers.

### Preparation of GO

GO was prepared by using the improved Hummers method [29, 30]. Briefly, graphite powder was oxidized using sulfuric acid, potassium permanganate, and hydrogen peroxide at a low temperature constant temperature water bath. Increased the temperature to 35 °C and continued the reaction for 2 h, then moved to a high-temperature reaction pot and added the appropriate amount of hydrogen peroxide and diluted hydrochloric acid to continue oxidation. Finally, the final graphene oxide after dialysis to neutral, ultrasonic stripping, centrifuge and freeze-dried was obtained.

### Preparation of MOF-5 and GO@MOF-5

**Fig. 1:**
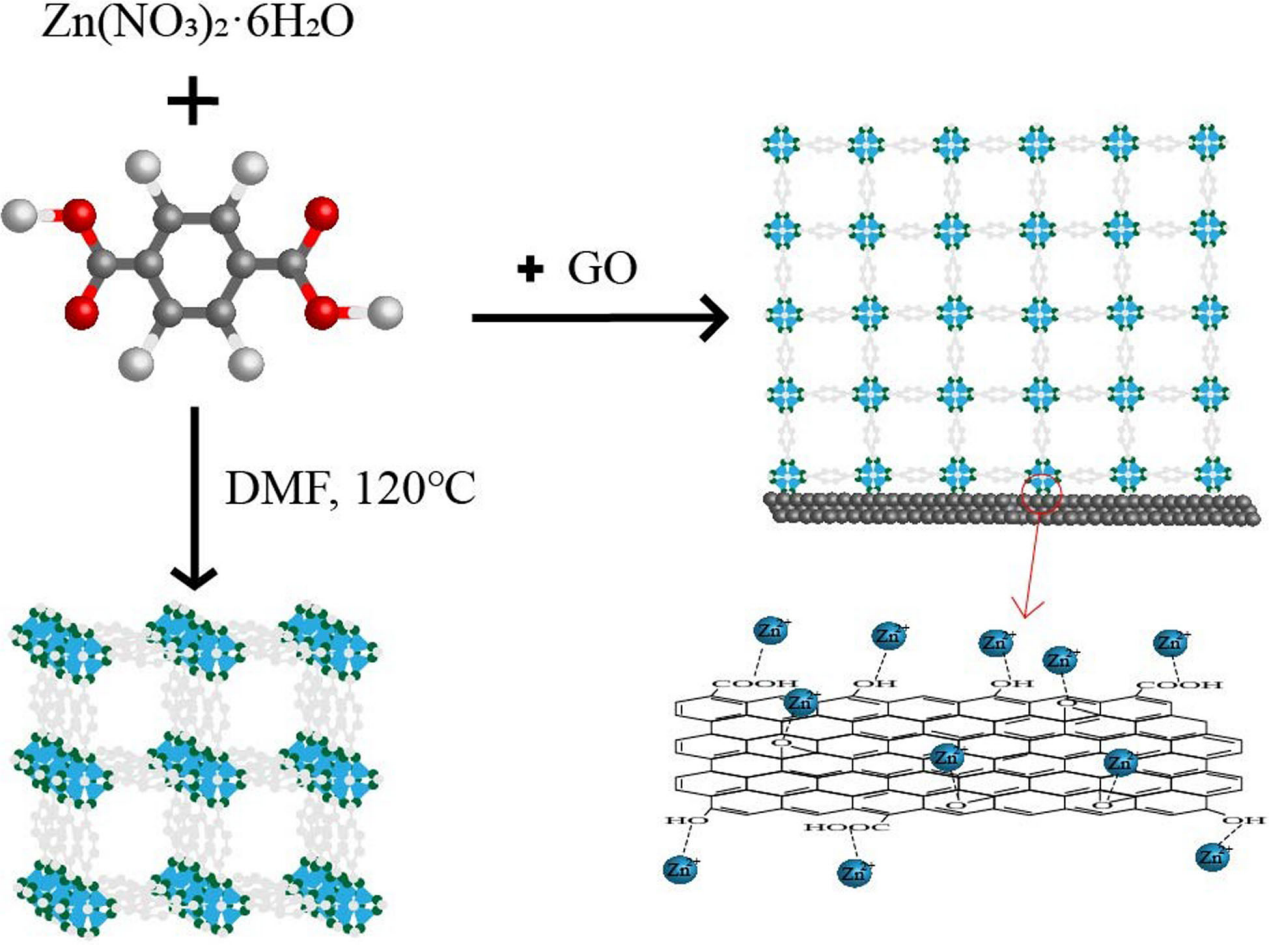
Preparation of (GO@) MOF-5

As shown in Fig. 1, zinc nitrate hexahydrate and terephthalic acid were dissolved in 30 mL of DMF in a high-temperature resistant reactor. The reaction mixture was heated in a furnace at 120 °C for 20 h to yield large cubic crystals of MOF-5. The reaction vessel was then removed from the furnace and cooled to room temperature. The cubic crystals were repeatedly washed with DMF and soaked in chloroform for 12 h, filtered, and dried.

The previously prepared GO was added to the precursor solution of MOF-5 and ultrasonically dispersed, and the remaining steps were in agreement with the preparation of MOF-5. In order to better explore the synergy between GO and MOF, 3.5%, 5%, and 7% GO ratios were added, respectively, and named MGG-3.5%, MGG-5%, and MGG-7%.

### Preparation of MOF-5/AP and GO@MOF-5/AP

Prepared MOF-5/AP using the solvent-nonsolvent method and chose DMF and ethyl acetate as solvent and nonsolvent respectively. First, the prepared (GO@) MOF-5 was added to 100 mL of ethyl acetate solution and stirred uniformly. Then took 10 mL as-prepared solution of AP (saturated solution of AP made from DMF as solvent), uniformly dropped the ethyl acetate solution mixed with (GO@) MOF-5 to the as-prepared solution of AP, and evenly stirred with a magnetic stir bar for 20 min. Finally, filtered and dried at 50 ^°^C for 12 h, and the mixtures of (GO@) MOF-5/AP were prepared.

### Characterization

The surface morphology, mean size, and size distribution of the prepared samples were characterized using scanning electron microscopy (SEM, SU-8020, Hitachi, Japan). A DX-2700 X-ray diffractometer (XRD, Dan Dong Hao Yuan Corporation, Liaoning, China) was used to analyze the element’s content of samples at a voltage of 40 kV and a current of 30 mA using Cu-Ka radiation. The structure was also analyzed by Fourier transform infrared spectrometer (FTIR-650, Tianjin Gangdong Corporation).

The Brunaure-Emmett-Teller (BET) surface area of as-synthesized samples was determined by physisorption of N_2_ at 77 K using a Belsorp-max surface detecting instrument.

The samples were analyzed using the DSC-131 differential scanning calorimeter (France Setaram Corporation, Shanghai, China). The conditions of DSC were as follows: sample mass, 0.5 mg; heating rate, 5, 10, 15, 20 °C/min; nitrogen atmosphere, 30 mL/min. Mettler Toledo TG thermal analyzer at a heating rate of 10 °C/min under nitrogen atmosphere.

Impact sensitivity type 12 drop hammer apparatus to test the impact sensitivity. The special height (*H*_50_) represents the height from which 2.5 ± 0.002 kg drop hammer will result in an explosive event in 50% of the trials. Test conditions for the dose were 35 ± 1 mg, temperature of 10~35 °C, relative humidity≦ 80%.

## Results and Discussion

### Characteristics of MOF-5 and GO@MOF-5

**Fig. 2:**
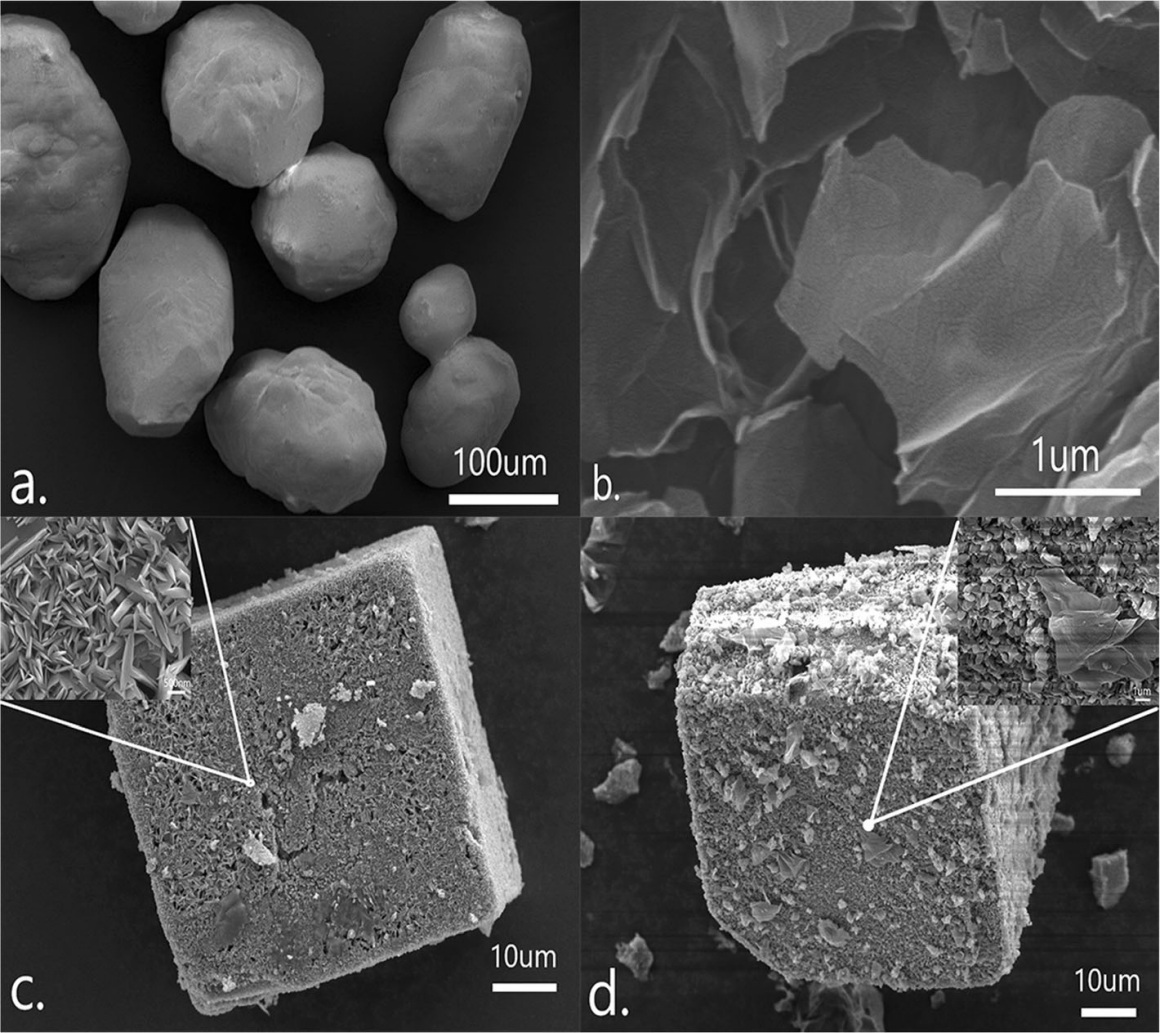
SEM images of (**a**) pure AP; (**b**) GO; (**c**) MOF-5, (**d**) GO@MOF-5

The texture of the samples can be observed on scanning electron microscopy (SEM) images presented in Fig. 2. Figure 2a showed a plot of AP material with different shapes, with an average particle size of about 100–200 μm. The surface of the GO has a partial wrinkle, and the edge is also stepped, which is generally a layered structure. It is clear that MOF-5 exhibits a regular cubic structure. The presence of pores can be observed at magnification, while GO@MOF-5 remains the cubic structure of MOF-5 and with many sheet-like GO attached to the surface, which also indicates that the addition of a small amount of carbon material does not affect the morphology of the MOF material

**Fig. 3.**
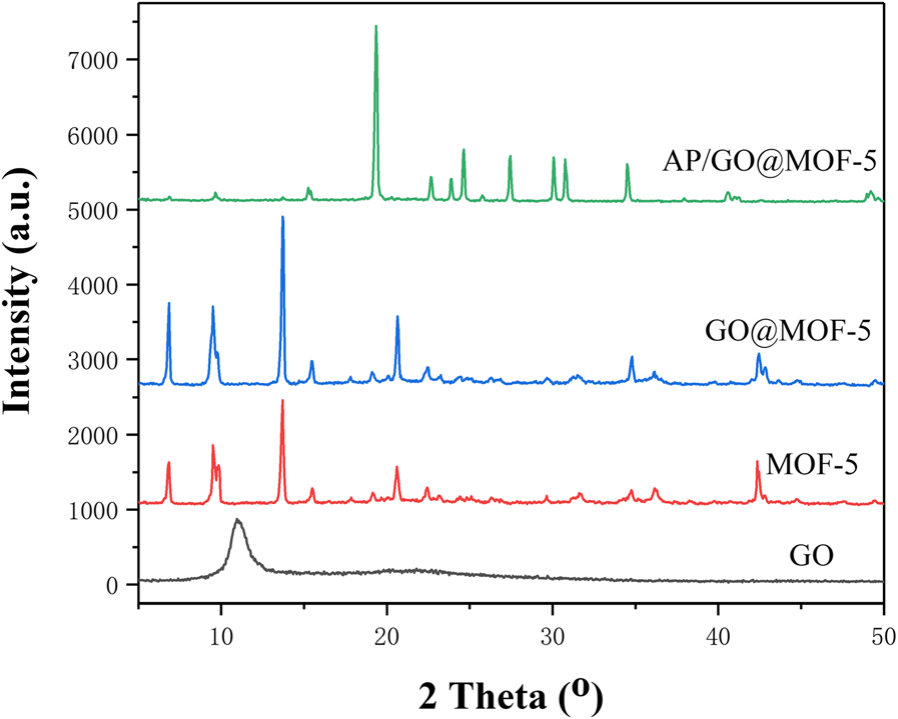
XRD curves of samples

Figure 3 showed the powder X-ray diffraction (XRD) of the synthesized composites. It can be clearly seen from the figure that the broad characteristic peak of GO at 2*θ* = 10.5^°^ indicates that the graphite structure has been destroyed and the interlayer spacing is enlarged to form a new structure. The peak position of the MOF-5 crystal is generally consistent with that reported in the literatures [25, 31]. Since MOF-5 is the host material, its structural characteristics occupy a dominant position in the composite material. The diffraction peak of the composite material can be seen from the XRD pattern and it basically coincides with the position of MOF-5. Petit et al. [32] reported that the peak of the composite material was split and became more and more obvious with the increased of GO. It was suspected that it may be due to the presence of GO, which increased the deformation of the material. However, in this study, it almost can be determined that there will be no significant effect on the structure of the material due to the small content of GO, which is consistent with the guess in the SEM analysis.

**Fig. 4.**
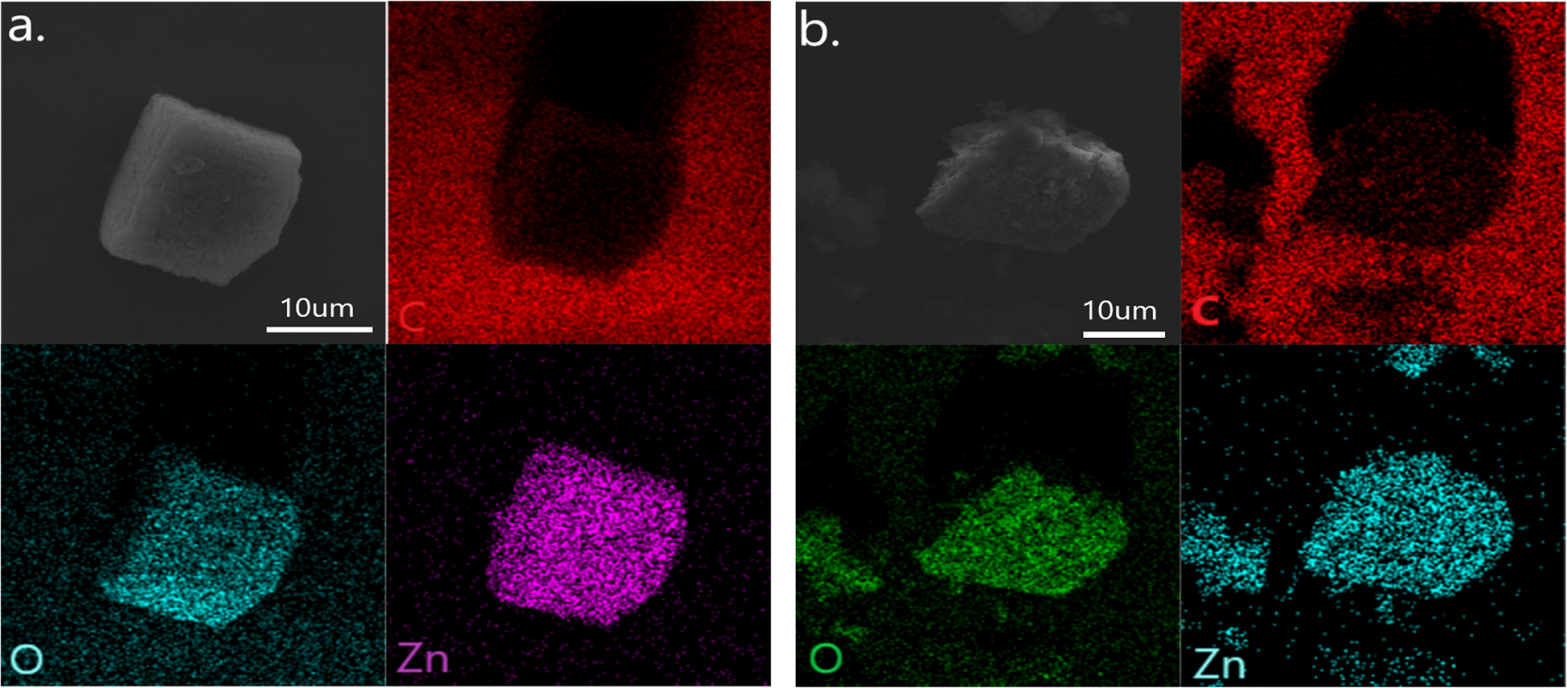
EDS mapping images of **a** MOF-5 and **b** GO@MOF-5

Energy dispersive spectroscopy (EDS) analysis was carried out to identify the components of MOF-5 and GO@MOF-5. Figure 4a showed the elemental mapping images of the uniform distribution of C, O, and Zn elements, which proves that the skeleton function of MOF material can make the corresponding metal elements evenly distributed and avoid the agglomeration of metal particles. And the elemental mapping images of GO@MOF-5 also showed the uniform distribution of C, O, and Zn elements. The carbon content is more obvious on the surface of the sample than MOF-5, indicating that the GO layer is well attached to the surface of the MOF material.

**Fig. 5.**
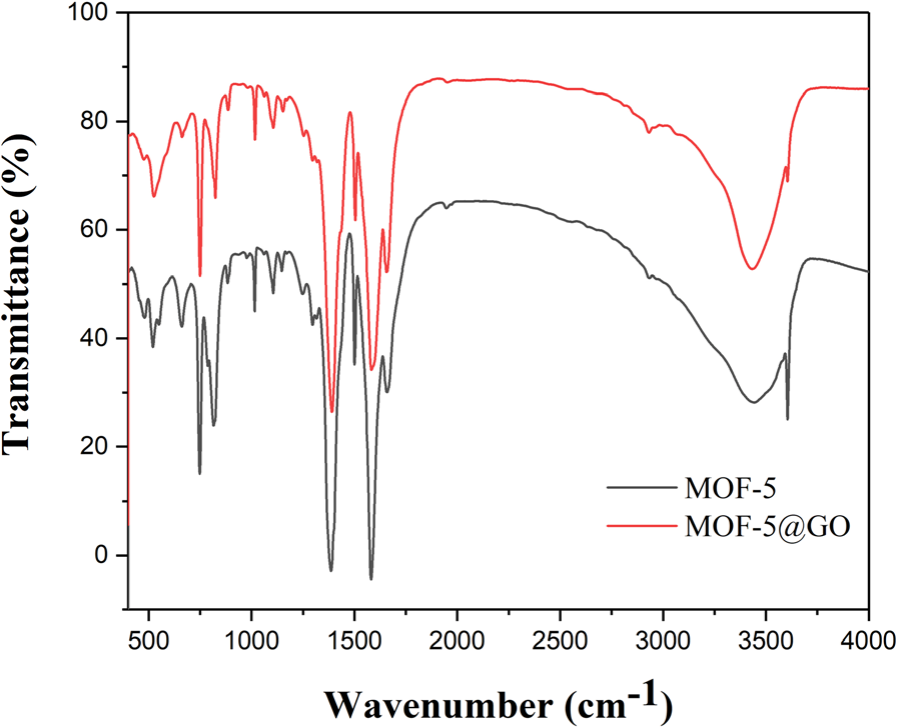
FT-IR spectra of MOF-5 and GO@MOF-5

Figure 5 showed the FT-IR spectra of MOF-5 and GO@MOF-5; the infrared spectrum of MOF-5 and its complex with carbon material are similar to those reported in related literature [20, 31]. As shown in the figure, the peak caused by symmetric stretching of carboxyl group in BDC can be seen at 1386 cm^−1^, and an asymmetric stretching peak of carboxyl group appears at 1581 cm^−1^. In addition, the broadband at 3000–3604 cm^−1^ guess may be the presence of water in the metal coordination. After adding a small amount of GO, the crystal structure of the composite remained essentially intrinsic, and there was no significant change. It was also proved that the addition of a small amount of GO did not affect the properties of MOF.

**Fig. 6.**
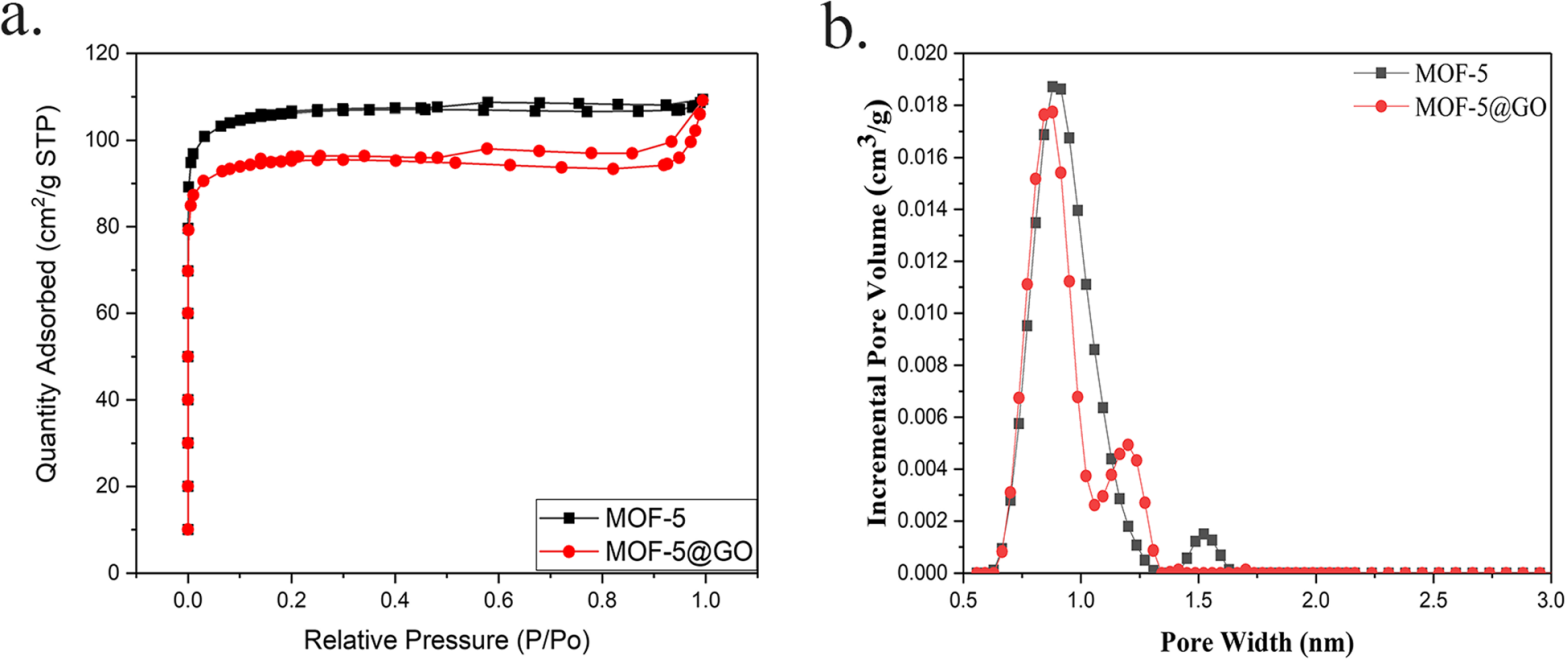
**a** Nitrogen adsorption isotherms and **b** incremental pore volume curves of MOF-5 and GO@MOF-5

The pore characteristics of samples were determined from the nitrogen adsorption isotherms which are shown in Fig. 6. The isotherm (Fig. 6a) reveals that the MOF samples exhibit typical type I sorption behavior. As derived from the N_2_ adsorption data, the Brunauer-Emmett-Teller surface area of MOF-5 is higher than GO@MOF-5, which was also reported by Petit [32]. When the content of GO is less than 10%, the specific surface area will decrease with respect to the MOF-5 raw material, which may be due to the blockage of some small pores when the GO is combined with the MOF, resulting in a decrease in BET. And it also indicated that the role of GO in the AP composite may depend mainly on its excellent thermal and electrical conductivity. It can be seen from Fig. 6b that the pore size distribution of MOF-5 and its composites are less than 2 nm, which proves that both materials belong to the microporous structure and are mainly concentrated in about 1 nm. Specific relevant parameters are clearly listed in Table 1.

**Table 1:** Nitrogen adsorption properties of MOF-5 and GO@MOF-5

Sample	BET surface area (m^2^/g)	Langmuir surface area (m^2^/g)	Pore volume(cm^3^/g)	Average pore diameter (nm)
MOF-5 GO@MOF-5	421.8908	464.9237	0.165578	1.56986
	384.5582	409.8701	0.148556	1.54521

### Influence of MOF-5 and GO@MOF-5 on thermal decomposition of AP

#### Thermal Analysis of Pure AP Particles

**Fig. 7.**
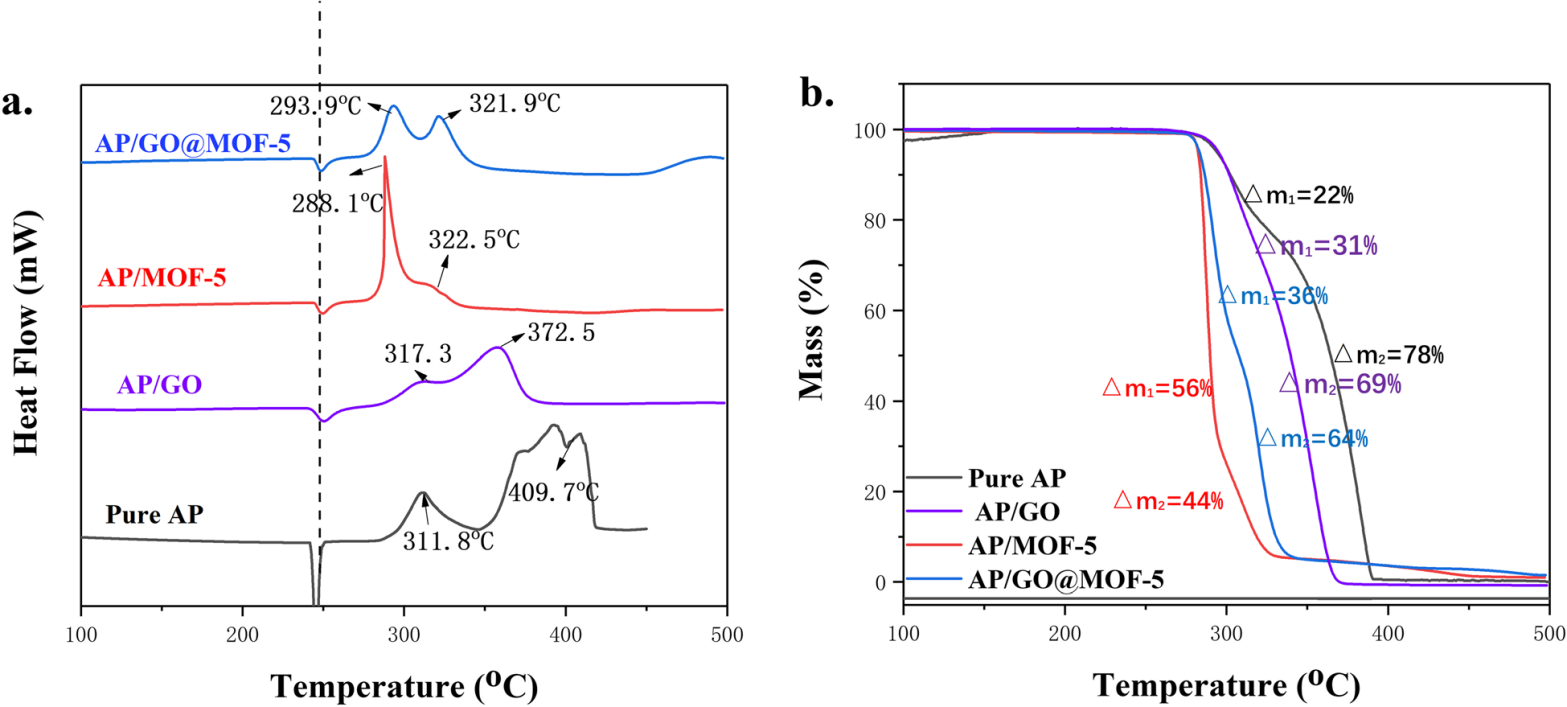
**a** DSC curves and **b** TG curves of AP composite samples (10 °C/min, N_2_ atmosphere)

The DSC curves of pure AP is shown in Fig. 7. The thermal decomposition of AP is a continuous and complex reaction process [4–18]. It can be seen from the figure that the curve has a distinct downward endothermic peak at 245 °C, which is the endothermic peak of the crystal transition of AP. At this time, the AP particle changed from orthorhombic to cubic, and the process is accompanied by endothermic. Then, there will be two upward exothermic peaks, which are low-temperature decomposition peak (LTD) at 311.8 ^°^C and high-temperature decomposition peak (HTD) at 409.7 °C. The low-temperature decomposition usually accompanied by dissociation and sublimation processes, mainly solid-phase reactions, and a small amount of gas-phase reaction [3]. During the decomposition of low temperature, AP begins to decompose and forms oxidative intermediates such as ClO, ClO_3_, O_2_, and H_2_O, and a part of O_2_ can be converted into superoxide ion (O_2_^−^), which will contribute to the oxidation of NH_3_, while unoxidized excess NH_3_ will adhere to AP crystal which hinders the thermal decomposition of AP, and the low-temperature decomposition process ends. As the temperature gradually increases, the excess NH_3_ desorbs, and the potential reaction centers on the surface of the AP crystal are reactivated, entering the high-temperature decomposition stage dominated by the gas-phase reaction until the AP is completely decomposed. The TG curve of pure AP also indicates that AP experienced two weight loss stages during thermal decomposition, resulting in mass loss of 22% and 78%, respectively, demonstrating the release of gaseous products corresponding to the two decomposition stages.

#### Thermal Analysis of AP/(GO@)MOF-5 Composites

It can be seen from Fig. 7a that the two exothermic decomposition peaks of the AP complex after the addition of the (GO@) MOF material are significantly advanced, while the first endothermic peak appeared at the same position as that of pure AP, showing that the catalysts have no influence on the crystal transition temperature. After the addition of GO, the pyrolysis stage is advanced, mainly due to the excellent heat transfer performance of GO and the high electron mobility exhibited during electron transfer. And the two decomposition peak temperatures of AP/MOF-5 were advanced to 288.1 °C and 322.5 ^°^C, respectively, while the decomposition peak temperature of AP/GO@MOF-5 was advanced to 293.9 ^°^C and 321.9 ^°^C, and the HTD peaks of the two composite materials were nearly 87 ^°^C ahead of AP raw material, indicating obviously catalytic effect on AP thermal decomposition. At the same time, it can be seen from the TG curves of Fig. 7b that the percentage of the first weight loss phase of the AP composite with (GO@) MOF material increased significantly, indicating that the thermal decomposition stage was shifted to a lower temperature, which is also reflected by the DSC curves. It also can be clearly seen from the DSC chart that the LTD stage is more obvious than the HTD and the peak area is larger, indicating that the HTD stage is almost simultaneously with the LTD stage.

**Fig. 8.**
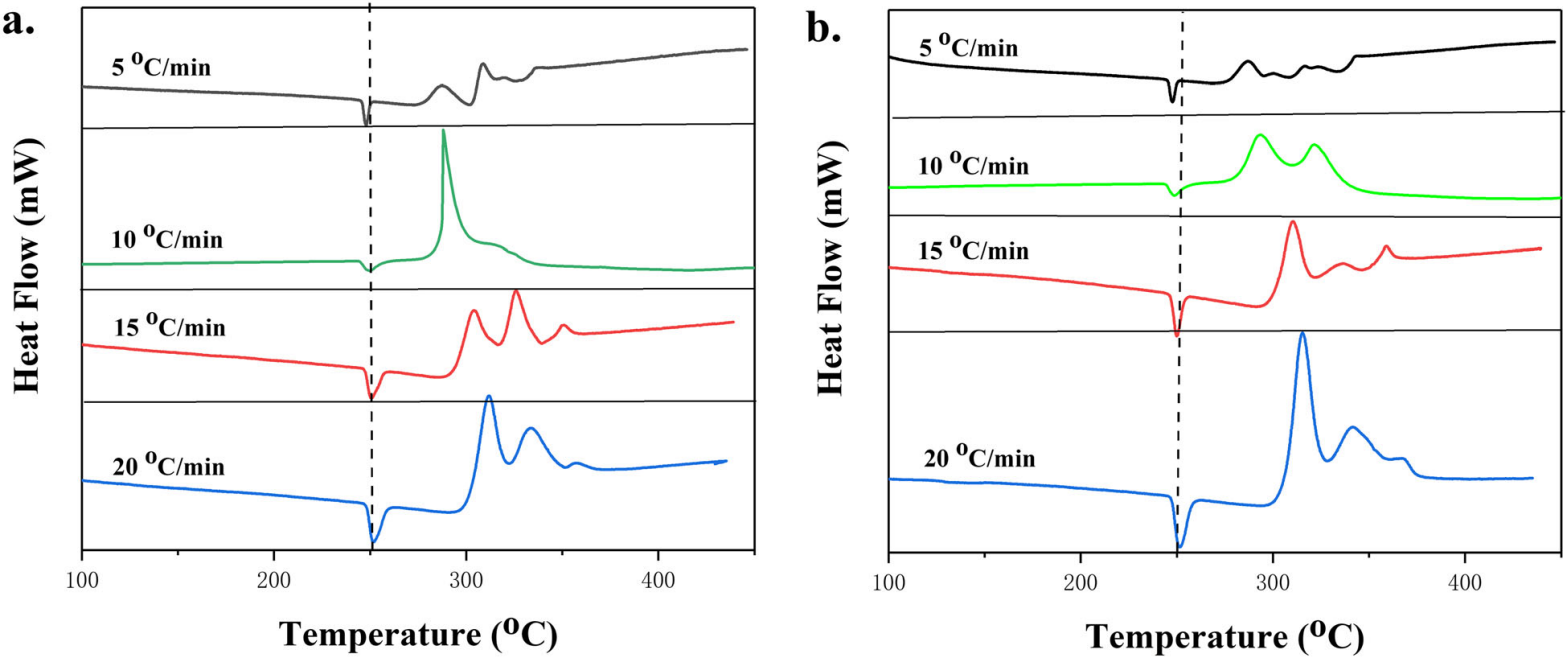
The effect of heating rate on DSC results of **a** AP/MOF-5 and **b** AP/GO@MOF-5 (N_2_ atmosphere)

In order to better explore the catalytic effect of (GO@) MOF composites on AP, the Kissinger method was used to determine the relevant kinetic parameters from the thermal data, and further demonstrating the catalytic effect. The activation energy (Ea) was calculated by testing peaks of DSC curves at different heating rates. The pre-exponential factor (A) can be obtained assuming that the decomposition follows first-order kinetics [3, 14]. The DSC curves of AP/MOF-5 and AP/GO@MOF-5 at different heating rates from 5 ^°^C to 20 ^°^C are shown in Fig. 8a, b, respectively. It can be seen from the figure that the LTD and HTD peaks of the samples are all transferred to higher temperatures by changing the heating rate from 5 to 20 ^°^C/min, which also indicates that the heating rate also has an effect on the decomposition process because that the sample has hysteresis to temperature at different heating rates. The relationship between decomposition temperature and heating rate can be described by Kissinger correlation [33].

1 $$ \ln \left(\frac{\beta }{T\mathrm{m}2}\right)=\ln \left(\frac{AR}{Ea}\right)-\frac{Ea}{RTm} $$

**Fig. 9.**
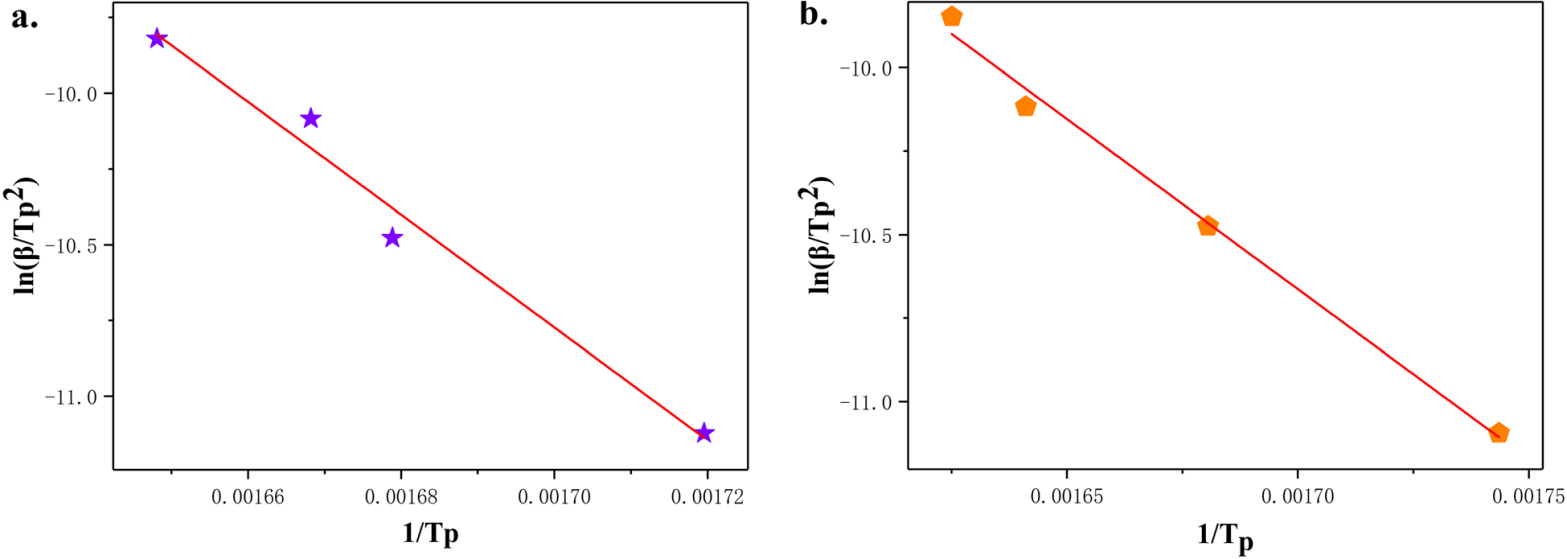
Fitting results of ln(β/T_p_^2^) and 1/T_p_ of **a** AP/MOF-5 and **b** AP/GO@MOF-5

**Fig. 10:**
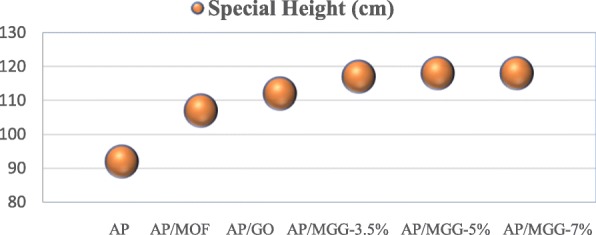
Impact sensitivity of samples

In this equation, where β, *T*_m_, *R*, *A*, and *E*_a_ are the heating rate/^°^C min^−1^, temperature/K, ideal gas constant, pre-factor, and activation energy/kJ mol^−1^, respectively. *E*_a_ can be calculated from the slope of the linear relationship between ln(β/*T*_m_^2^) and 1/*T*_m_. Figure 9 showed the linear fit of ln(β/*T*_m_^2^) and 1/*T*_m_ of AP/MOF-5 and AP/GO@MOF-5, demonstrating that the thermal decomposition of these samples follows the first-order kinetic law and is calculated specifically, and the data are shown in Fig. 10. The enthalpy of the AP compound has a significant increase from 576 J g^−1^ to 815.9 J g^−1^ and 1011 J g^−1^ after the addition of the (GO@) MOF material and the *E*_a_ decrease from 143.8 kJ mol^−1^ to 139.6 kJ mol^−1^ and 84.6 kJ mol^−1^ accordingly, which may be due to the thermodynamics of some metal oxides or chlorides formed during the catalytic process is unstable, which reduces the *E*_a_ of AP thermal decomposition.

At the same time, in order to better prove the role of GO in the AP composite, the special height (*H*_50_) of the AP-based composite samples were tested, and the test results are shown in Fig. 10. It can be seen from the table that the sample with the GO@MOF material has the tallest value of *H*_50_, which means its sensitivity is lower than other samples, indicating that the addition of GO effectively exerts its excellent thermal conductivity and alleviates the local heat that may lead to the generation of “hot spots,” and it does improve the stability and security of the entire system. In addition, with the increase of GO content, the stability of the system has also been subtly improved, which also confirms the effect of GO.

**Fig. 11.**
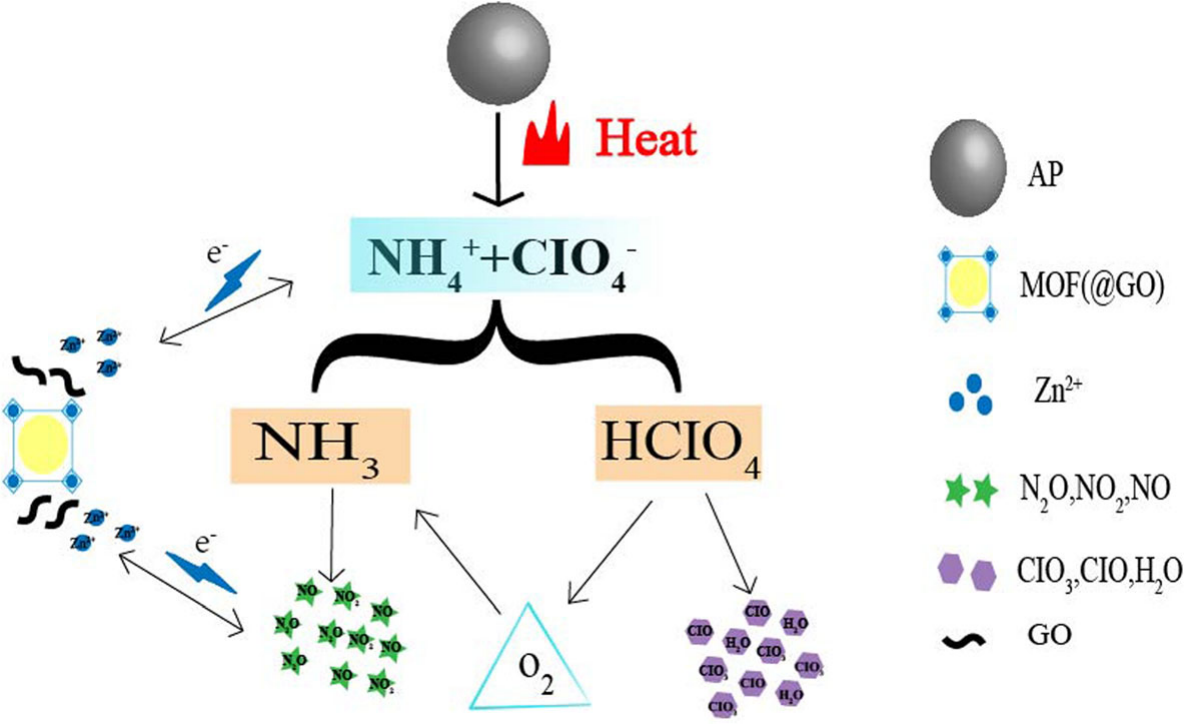
Mechanism diagram of AP/(GO@)MOF thermal decomposition

From the above results, it is clear that (GO@) MOF can significantly promote the thermal decomposition process of AP, especially the high-temperature decomposition and enthalpy. The presence of ammonia and chlorine is often the main reason for delaying the thermal decomposition of AP, namely, the decomposition of perchloric acid and the oxidation of ammonia [3]. The MOF material can effectively adsorb gaseous ammonia molecules, prevent it from adsorbing on the surface of the AP crystal to promote the decomposition reaction and decrease the decomposition peak temperature. At the same time, as shown in Fig. 11, the MOF-loaded Zn is in an unsaturated state, and it is easy to adsorb substances with excess electrons, especially easy to break the N-X bond, resulting in an increase in ΔH. And it is also easily reacted with nitrogen oxides generated by AP decomposition, which can prompt the rapid decomposition reaction. Besides, the transition metal ion can also accelerate the conversion of O^2^ produced by the decomposition of HCIO_4_ into superoxide ion (O_2_^−^), contributing to the oxidation of NH_3_. In the whole thermal decomposition process of AP, GO mainly takes advantage of its high thermal conductivity and electrical conductivity, and effectively reduces the formation of hot spots, thereby reducing sensitivity and improving system stability.

**Fig. 12:**
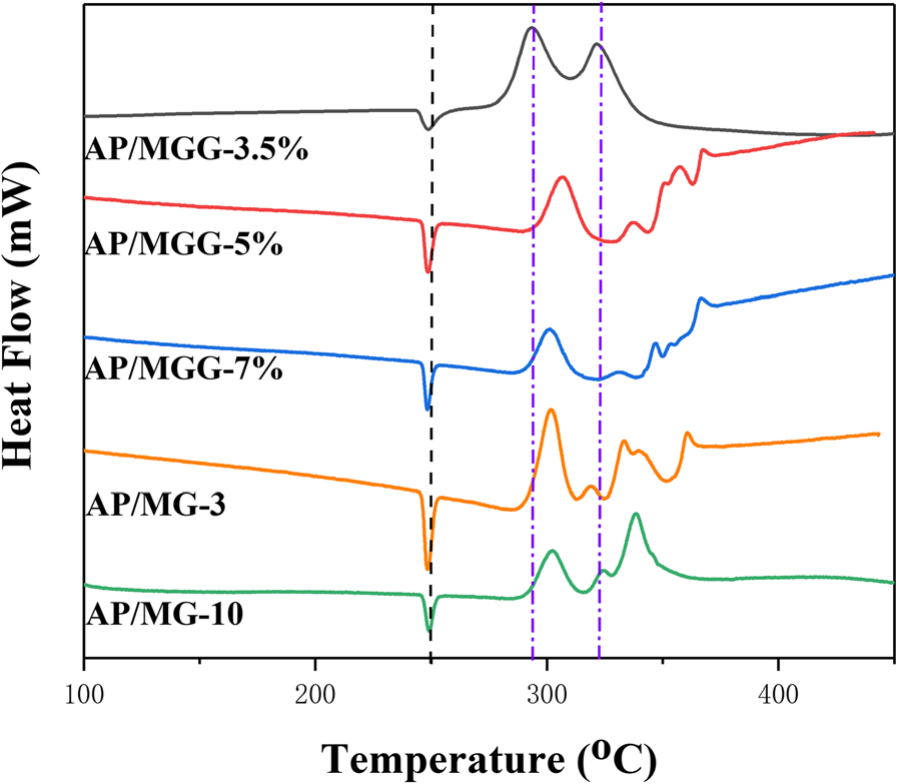
DSC curves of different proportions of samples (10 °C/min, N_2_ atmosphere)

**Fig. 13:**
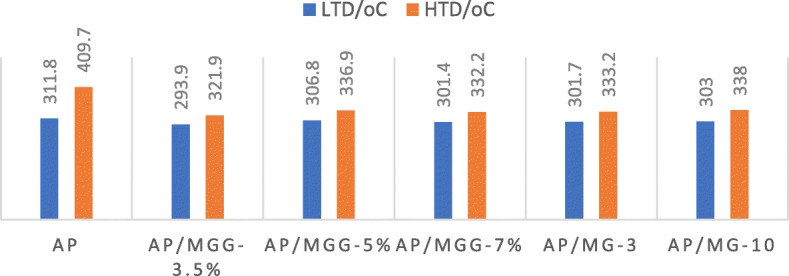
Thermodynamic parameters of different proportions of samples

In addition, attempts have been made to change the ratio of MOF materials and the proportion of GO in GO@MOF composites. The specific DSC curves and related data are shown in Fig. 12 and Table 2. In order to distinguish different samples better and easier, AP/MOF-5 with 3% and 10% MOF is referred to as AP/MG-3, AP/MG-10, respectively. MOF accounts for 5%, while GO accounts for 3.5%, 5%, and 7% and are abbreviated as AP/MGG-3.5%, AP/MGG-5%, and AP/MGG-7%. It can be seen from the DSC curves of Fig. 9 that when the GO content is 3.5% and the ratio of GO@MOF in the AP composite is 5%, the two decomposition peak temperatures are most advanced, and the corresponding catalytic effect is also optimal (Fig. 13).

**Table 2:** Thermodynamic parameters of samples at different heating rates

Sample	Heating rate/^°^C min^−1^	LTD/^°^C	HTD/^°^C	△H/J g^−1^	Ea/kJ mol^−1^
AP	5	303.4	402.3	576 [34]	143.81
	10	311.8	409.7		
	15	316.2	425.7		
	20	323.7	434.8		
AP/MOF-5	5	287.6	306.2	815.8	139.60
	10	288.1	322.5		
	15	303.9	326.3		
	20	311.6	333.6		
	5	303.4	354.2		
AP/GO	10	317.3	372.5	937.2	94.68
	15	326.9	384.3		
	20	336.1	401.2		
AP/GO@MOF-5	5	286.8	300.4	1011	84.65
	10	293.9	321.9		
	15	310.8	336.2		
	20	315.5	342.2

## Conclusions

In summary, AP/(GO@) MOF composites were prepared, and their catalytic effects on AP thermal decomposition were investigated. The experimental results showed that GO@MOF composites have a significant catalytic effect on the thermal decomposition of AP, and not only the HTD of AP is significantly advanced, but also the ΔH is effectively increased and the activation energy of the reaction is lowered. Among them, when the GO content is 3.5% and the total content of GO@MOF-5 is 5%, the AP complex has the best catalytic effect. This is mainly due to the large specific surface area of the MOF material, which provides more reactive centers and its strong adsorption, as well as the great advantage of GO in heat conduction and electron transfer, which synergistically accelerates the thermal decomposition process of AP. In addition, this study provides experimental and theoretical support for the application of MOF and GO composites in AP thermal decomposition.

## Abbreviations

AP: Ammonium perchlorate
BET: Brunauer-Emmett-Teller
CCSs: Carboxyl-rich carbon spheres
CNT: Carbon nanotube
DSC-TG: Differential scanning calorimetry and thermalgravimetric
*E*a: Activation energy
EDS: Energy dispersive spectroscopy
FT-IR: Fourier transform infrared spectrometer;
GO: Graphene oxide
H2BDC: 1,4-benzenedicarboxylic acid
HTD: High-temperature decomposition
LTD: Low- temperature decomposition
MOF-5: Zn4O(benzene-1,4-dicarboxylate)3
MOFs: Metal-organic frameworks
SEM: Scanning electron microscopy
XRD: X-ray diffraction
ΔH: Enthalpy
